# Particle-Bed Binding by Selective Paste Intrusion—Strength and Durability of Printed Fine-Grain Concrete Members

**DOI:** 10.3390/ma14030586

**Published:** 2021-01-27

**Authors:** Daniel Weger, Christoph Gehlen

**Affiliations:** Chair of Materials Science and Testing, Centre for Building Materials (CBM), Technical University of Munich, 81245 Munich, Germany; gehlen@tum.de

**Keywords:** 3D printing, additive manufacturing, particle-bed, selective binding, particle-bed binding, selective paste intrusion, fine-grain concrete, cement, cementitious material, strength, durability

## Abstract

The selective paste intrusion (SPI) describes a selective binding, additive manufacturing method. SPI bonds thin layers of aggregate by cement paste locally. Currently, SPI can achieve higher compressive strength, durability, and easier unpacking behavior compared to other selective binding methods suitable for the production of concrete structures. Particle-bed based methods not only achieve much higher surface resolutions than depositing (extrusion)-based additive manufacturing methods but also have no restrictions in freedom of form. However, the mechanical performance of SPI components strongly depends on the void content between the individual layers and thus the penetration behavior of the cement paste. This paper presents direction-dependent measurements of the strength and durability of SPI-printed components compared to casted specimens with the same mixing composition. The results show compressive strength values between 70 and 78 MPa after 7 d, flexural strength of 1/10 without reinforcement, a high freeze–thaw resistance, no detectable carbonation after 182 days of exposure under ambient CO_2_–conditions, and after 28 days under increased CO_2_ content of 2 vol % as well as low chloride penetration resistances. All tests showed in almost all cases no dependency on the layer orientation.

## 1. Introduction

Beginning in the 1980s, additive manufacturing (AM) processes are increasingly revolutionizing the production of complex-shaped elements [1,2,3,4,5]. While particle-bed binding (3D printing) is already state of the art in many fields of production, it is still in state of research for civil engineering applications. In 1995, Pegna was the first to apply a particle bed binding process in combination with cement as a binder [6,7]. Particle-bed-based methods for cementitious materials (concrete construction) use all the same basic principles. Thin layers of particles are spread and subsequently locally bonded by an activator or a binder. In addition to the material extrusion methods (e.g., concrete printing and contour crafting) and material jetting, Buswell et al. [8] as well as Lowke et al. [9] define three particle-bed binding methods: the selective cement activation (SCA), selective paste intrusion (SPI), and binder jetting. However, only SCA and SPI can be used for the direct production of concrete elements.

The SCA (binder: cement or geopolymer) uses thin layers of aggregate–binder mixtures which are solidified by a water-based activator that leads to very fine surface resolutions [9,10,11]. Controlling the water penetration behavior and the water distribution between the layers is crucial for the strength and shape accuracy of the constructions [9,12,13,14,15,16]. The SCA method has already been in focus for building lunar outposts [17] and can be used for the production of structures made of lightweight concrete, too [18].

SCA-produced specimens show a dependency of strength on the layer direction and the post processing. The following examples show exemplary compressive strength values.

In 1997, Pegna [7] presented a compressive strength of 33.8 MPa in 90° to the layer direction and 28.3 MPa in 0° by testing specimens with a size of 16 × 16 × 15 mm^3^ and using Portland cement as binder. Cesaretti et al. [17] used a combination of MgO and MgCl_2_ (Sorel cement) and achieved a compressive strength of 20.3 MPa in 90°. Fromm [11] (again using Portland cement as binder) showed compressive strength values of 13.4 MPa in 90° and 10.0 MPa in 0° with a specimen size of 10 × 10 × 10 mm^3^ after 28 d post processing under water and another 2 d drying at 60 °C. In 2015 and 2020, Lowke et al. determined compressive strength of 16.4 and 15.4 MPa using a specimen size of 40 × 40 × 40 mm^3^ without further post processing but different production processes. However, both investigations showed an increasing compressive strength with increasing water content. This could be confirmed by the process technological effect of the water distribution between the layers, which could exemplary be shown in [9]. This was confirmed by the results of Shakor et al. [19] (8.3 MPa in 90°, specimens 20 × 20 × 20 mm^3^) in 2017. Furthermore, in 2019, Shakor et al. [20] showed the effect of a post treatment with enhanced temperatures (40 °C drying before testing). There, a compressive strength of 14.7 MPa (dried) and 4.8 MPa (wet) was measured in a direction of 90° and using specimens with a dimension of 20 × 20 × 20 mm^3^. In 2020, Shakor et al. [16] presented comparative investigations of gypsum and cement mortar specimens. Again, the compressive strength of the cement increased by increasing water content up to 2.8 MPa.

Another group of researchers of the SCA method are using geopolymer binders [15,21,22,23,24]. In those investigations, the layer direction as well as the post processing showed also an important effect on strength. However, since the focus of the paper is on cementitious binders, these references are not described in detail.

The selective cement paste intrusion method (SPI) uses thin layers of aggregate that are bond by cement paste. In 2016, Weger et al. [25] showed results for the compressive strength of cylinders with a diameter of 50 mm and a height of 51 mm after 7 d without any further post processing of 22.1 MPa in 90°. Furthermore, in 2018, Prasittisopin et al. [26] achieved a compressive strength of 37.3 MPa in 90° and 33.8 MPa in 0°. In 2018, Weger et al. [27] exhibited layer-dependent results for the compressive strength of specimens with dimensions of 100 × 100 × 100 mm^3^ of 70.6 MPa at 90° and 64.2 MPa at 0°. Furthermore, a good durability (mainly caused by its high material density after printing), easier unpacking behavior compared other selective binding methods (mainly caused by the coarser particles used in the particle bed), and densities of normal concrete can be achieved [27,28,29]. In addition, a first concept for a 3D reinforcement was published [30].

However, the mechanical performance of SPI components strongly depends on the layer bonding as well as on the void content between the individual layers and thus the penetration behavior of the cement paste. The penetration depth of the cement paste is connected to its rheological properties and the flow resistance of the particle bed [29,31]. Therefore, in practice, time and cost-intensive trial and error tests are necessary to evaluate new cement paste–particle bed combinations. Therefore, the first publications focus on the modeling and simulation of the penetration behavior of the cement paste depending on the rheological parameters and properties of the particle bed [9,29,32,33,34,35].

Currently, SPI seems to be able to achieve higher compressive strength than other selective binding methods. On the other hand, the surface resolution of SCA is finer than that of SPI. The surface resolution of SPI is in between the material extrusion processes and SCA. However, compared to SCA, SPI can achieve a faster construction speed due to its coarser particles and its associated higher layer thickness by using a single nozzle.

**Figure 1:**
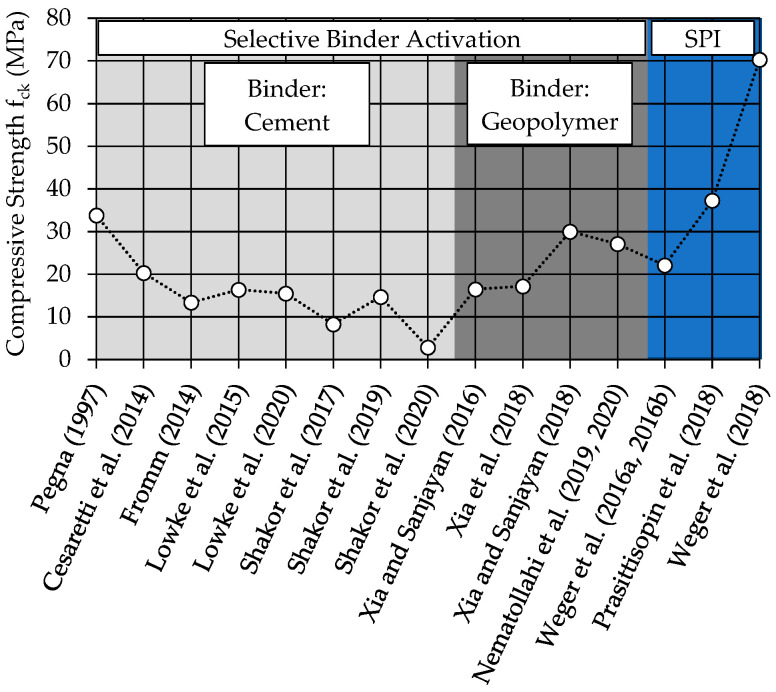
Comparison of compressive strength values at 90° for particle-bed-based methods [29].

However, it must be noticed that Figure 1 only shows compressive strength values at 90° to the layer direction. Furthermore, the specimen size (10 × 10 × 10 mm^3^ to 100 × 100 × 100 mm^3^), the age of the specimens, the used layer height, the printer technology, as well as the post treatment and curing varied. However, the maximum values published in the papers were represented.

Due to the good material properties (strength, durability, density) combined with a beneficial unpacking behavior and production speed, SPI seems to be a promising alternative for the additive manufacturing of cementitious material for application in construction. However, due to different material and process combinations (material composition, layer thickness, curing, etc.) the results published so far are not directly comparable. To prove whether SPI is truly applicable to construction, strength and durability must be tested for a material manufactured with the same process parameters.

Therefore, as a novelty, this paper presents a comprehensive characterization of strength (compressive and flexural strength) and durability (carbonation, chloride, and freeze–thaw attack) of SPI specimens produced with the same printing process and material combination. Furthermore, all investigations on 3D-printed specimens were done direction dependent (90° and 0°) and were compared to conventionally cast reference specimens.

## 2. Materials and Methods

### 2.1. Printing Process

**Figure 2:**
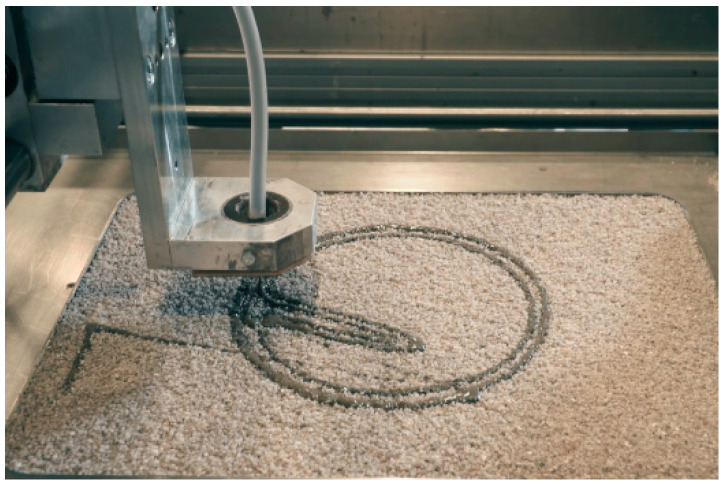
Selective paste intrusion (SPI) printer used for the investigations [36].

The printer used in this paper is equipped with an x-y gantry system with a building platform with dimensions of 0.305 to 0.375 m; see Figure 2. The building platform can be moved for 0.250 m in height. The gantry system uses a conical, round nozzle with an inner diameter b_nz_ of 2.0 mm (nz = nozzle), which moves with a height h_nz_ of 15.0 mm over the particle bed. A single particle layer height was 3.0 mm. The printing speed (movement) of the nozzle was 2000 mm/min with a volume output of cement paste of 2.22 × 10^−4^ m^3^/m. The outflow of the cement paste was adjusted so that the cement paste was continuously applied to the particle bed with as little pressure as possible; see also [35].

**Figure 3:**
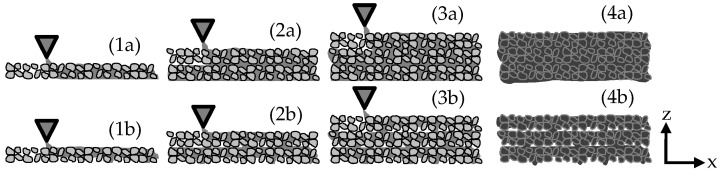
Production process with complete layer bonding/filling of voids (1a)–(4a) and with incomplete layer bonding/filling of voids (1b)–(4b) [28].

The cement paste is pumped from a reservoir to the nozzle by a peristaltic pump controlled by a stepping motor. After the local application of the cement paste on the particle bed, the building platform on which the particle bed is spread is lowered by the set layer thickness, and a new particle layer is scattered (here 3 mm) and smoothed; see Figure 2 and Figure 3 (1a–4a). However, if the rheology of the cement paste is not adapted to the flow resistance of the particle bed, insufficient shape accuracy or layer bonding with voids will follow; see [25,28,29,31,32,37] and Figure 3 (1b–4b).

**Figure 4:**
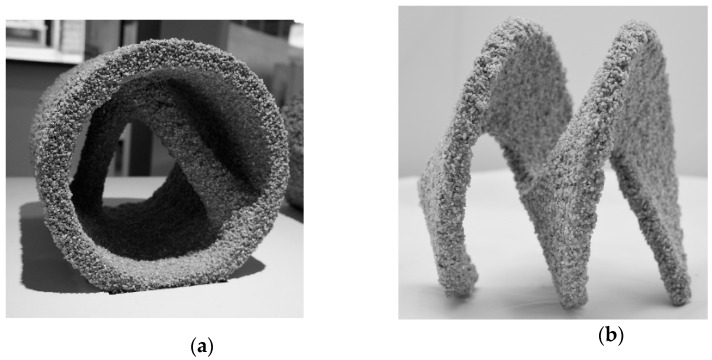
(**a**) SPI manufactured tube with internal double bracing; (**b**) SPI manufactured Helix (wing thickness 0.015 m), credit of picture (**b**): C. Matthaeus.

After the printing is finished, the component is excavated from the building space after 1 d to ensure a comparable curing to the casted specimens before exposition to laboratory conditions. However, depending on the cement paste composition, removal is possible already after a few hours [29,38]. A major advantage is the almost dust-free unpacking process and the good flowability of the unbound particles, which results in easy unpacking. The unbound aggregates can be re-used for a new print. Due to the support of the unbound particles, overhangs of almost any complexity can be realized; see Figure 4.

### 2.2. Materials

#### 2.2.1. Cement Paste

**Figure 5:**
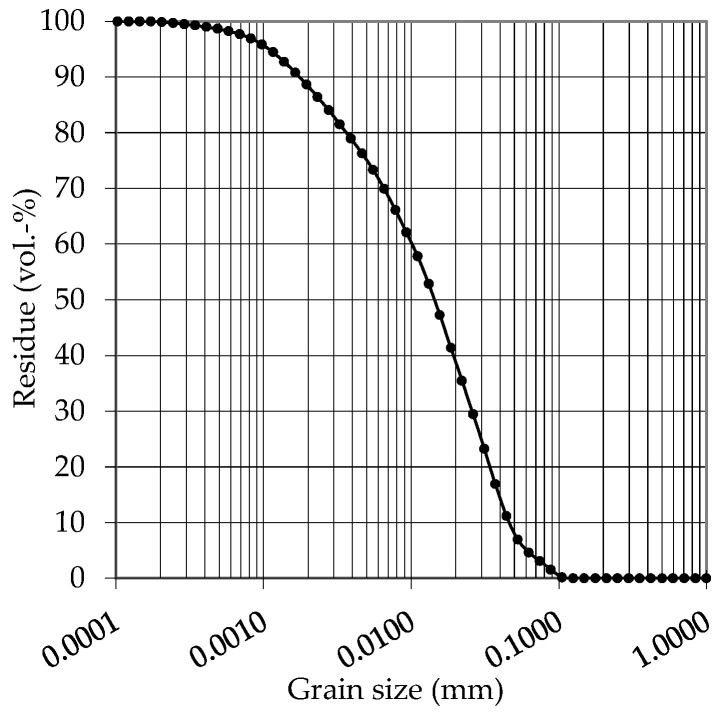
Grain size distribution of the used Ordinary Portland cement (OPC).

An Ordinary Portland cement (OPC, CEM I 42.5 R) was used as cement for all investigations. The grain size distribution of the cement can be taken from Figure 5.

Demineralized water was utilized as mixing water. The mixing water was pre-cooled to 1.5 °C using a cryostat in order to achieve a cement paste temperature of 20 ± 1 °C. Furthermore, a polycarboxylate ether-based superplasticizer (PCE) with a solid content of 35.1 wt % has been added to adjust the rheological properties of the cement paste for a sufficient penetration behavior. The water content of the PCE was charged in the mixing water.

The mixing process was as follows: First, the PCE was mixed into the mixing water. Then, the cement was placed in the mixer, and the water/PCE solution was added over the first 30 s of the mixing process. The first mixing section lasted 90 s, which was followed by a 120 s pause time in order to remove cement adhering to the bottom of the mixing container and to the mixing tool. Then, the mixing process was continued for another 90 s. The device used was an intensive mixer with a star-type rotor (, model R 02, company Eirich, Hardheim, Germany) at fastest speed (stage 2 of 2 for mixing tool and container).

For the investigations of this paper, a cement paste with a water to cement (w/c) ratio of 0.30 as well as mini slump flow (Haegermann cone, glass plate) of 400 mm with a PCE content of 0.720 wt % was used. The density of the fresh cement pastes was 2090 kg/m^3^. The yield stress of the cement paste, which was calculated using Herschel–Bulkley, was 2.7 Pa (consistency factor k = 0.12 Pa·s^n^ and flow index n = 1.26), and the thixotropy A_thix_ was 0.42 Pa/s. The rheological properties were determined using a double-plate geometry with a diameter of 50 mm and 1 mm gap size as well as using a vane-in-cup geometry for determination A_thix_; see Appendix A, Table A1 and [29,35].

#### 2.2.2. Aggregate

A sieved and fire-dried quartz sand with a grain size of 1.0–2.2 mm (d_50_ = 1.6 mm) was used. The density of the grains was 2643 kg/m^3^, the bulk density was 1447 kg/m^3^, and the porosity was 0.453.

### 2.3. Methods

#### 2.3.1. Casted Specimens

**Figure 6:**
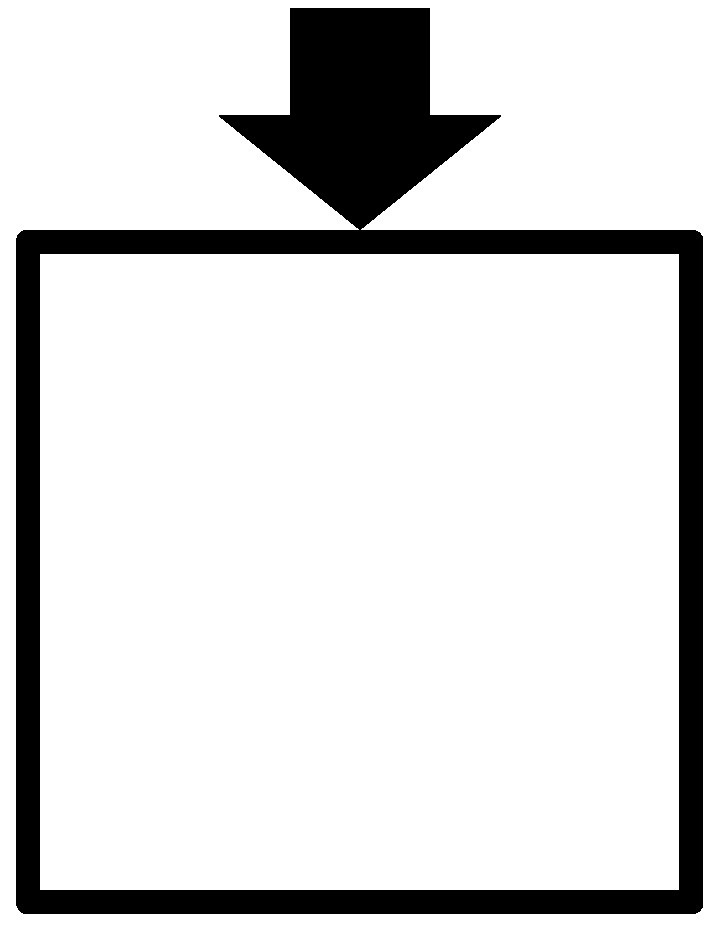
Casted reference specimen (REF) with independent load direction (black arrow) due to non-existent layering [28].

As a reference, conventionally mixed and casted specimens (REF) were prepared. The cement paste was mixed separately according to Section 2.2.1 and homogenized with the dry aggregate of Section 2.2.2 in a compulsory mixer for 90 s. The amount of cement paste added corresponded to the porosity between the aggregates. After filling the molds, which had previously been treated with formwork oil, the specimens were compacted on a vibrating table until no more air bubbles reached the surface. The reference specimens were tested independently of direction; see Figure 6.

#### 2.3.2. 3D-Printed Specimens

**Figure 7:**
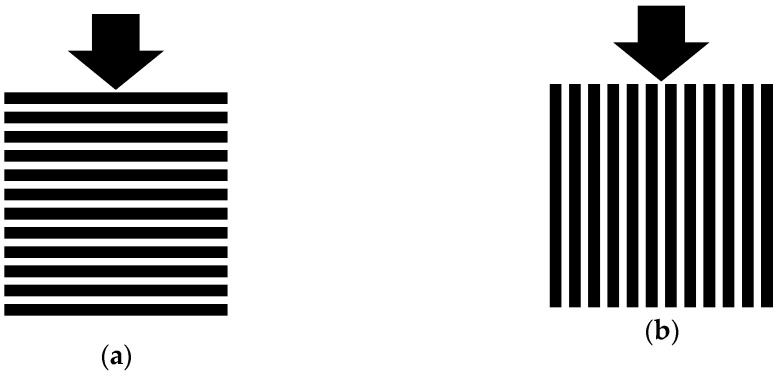
3D-printed specimens with load direction of (**a**) 90° and (**b**) 0° to the layers [28].

The 3D-printed specimens were produced with a layer thickness of 3 mm. The strength and durability properties have been tested in two directions: perpendicular to the layer of the specimens (90°) and parallel to the layers of the specimens (0°); see Figure 7.

Furthermore, for the compressive strength tests, the proportion of filled voids of the 3D-printed specimens was controlled via the applied volume of the cement paste per layer.

#### 2.3.3. Determination of the Air Void Content of the REF Specimens in Fresh State

The determination of the air void content of the fresh cement paste–aggregate mixture was carried out in accordance with the German standard DIN EN 1015-7:1998-12 [39] by means of the pressure equalization method in a 1 L container.

#### 2.3.4. Determination of the Density of Hardened Specimens

The density of the specimens was calculated by weighing according to the German/European standard DIN EN 12390-7:2009 [40].

#### 2.3.5. Determination of the Air Void Content of 3D-Printed Specimens

Since it is not possible to determine the density or the air void content in the fresh state of the 3D-printed specimens, the calculation of the voids must be carried out using a theoretical approach [28] using Equation (1).

(1) $$\rho_{\mathrm{spe}}=\rho_{a}\cdot \varphi +\rho_{P}\cdot \left(\varepsilon -\Phi_{\mathrm{TAC}}\right),$$

where $\rho_{\mathrm{spe}}$ is the density of the specimen in kg/m^3^, $\rho_{a}$ is the density of the aggregate in kg/m^3^, φ is the solid ratio, $\rho_{P}$ is the density of the cement paste in kg/m^3^, ε is the porosity of the particle bed, and $\Phi_{\mathrm{TAC}}$ is the total air void ratio of the specimen.

Equation (1) leads to Equation (2) to calculate the total air void content (TAC) in vol %.

(2) $$\mathrm{TAC}=\varepsilon -\frac{\rho_{\mathrm{spe}}-\rho_{a}\cdot \varphi }{\rho_{P}}\cdot 100$$

Furthermore, the filling ratio of the voids in the particle bed $\Phi_{\varepsilon }$ can be calculated following Equation (3).

(3) $$\Phi_{\varepsilon }=\frac{\varepsilon -\Phi_{\mathrm{TAC}}}{\varepsilon }=\frac{\frac{\rho_{\mathrm{spe}}}{\rho_{P}}-\frac{\rho_{a}\cdot \varphi }{\rho_{P}}}{\varepsilon },$$

There, when $\Phi_{\varepsilon }$ = 1, the voids of the particle bed are completely empty, and the density is the bulk density of the particle bed, and the $\Phi_{\mathrm{TAC}}$ or TAC is maximal as well [29].

#### 2.3.6. Determination of Compressive and Flexural Strength

The compressive strength was determined according to DIN EN 12390-3:2009-07 [41] on casted specimens as well as on 3D-printed specimens in 0° and 90°. For this purpose, cubes with a side length of 100 mm were produced conventionally in molds and were printed with a dimension of 100 × 100 × 120 mm^3^ depending on the loading direction. Then, the 3D-printed specimens were wet-sawn to the dimension of 100 mm side length. The loaded sides were grinded to ensure a plane-parallel test surface.

Furthermore, reference prisms (40 × 40 × 160 mm^3^) were casted in molds to examine the compressive and flexural strength according to DIN EN 196-1:2016-11 [42].

After printing, the specimens were left in the particle bed for 1 d and then stored at 20 °C and 65% relative humidity (RH) without any further post-treatment until the day of testing after 7 d.

After concreting, the casted specimens were left in the formwork for 1 d and stored at 20 °C and 65% RH until the day of testing after 7 d without any further post-treatment apart from grinding the test surface.

#### 2.3.7. Determination of the Freeze–Thaw Resistance

The freeze–thaw resistance of the casted reference and 3D-printed specimens in 0° and 90° was tested according to DIN CEN/TS 12390-9:2017-05 (DIN SPEC 91167) [43] with (CDF) and without (CIF) deicing salt. The casted reference specimens were produced in accordance to the standard in a cubic formwork with a side length of 150 mm with the test surface molded on Teflon. Furthermore, specimens with the dimensions 160 × 160 × 70 mm^3^ were printed.

The conventionally produced specimens remained in the formwork and the SPI specimens in the particle bed both for 1 d. After that, the specimens were stored under water until day 7 and then until day 28 at 20 °C and 65% RH.

**Figure 8:**
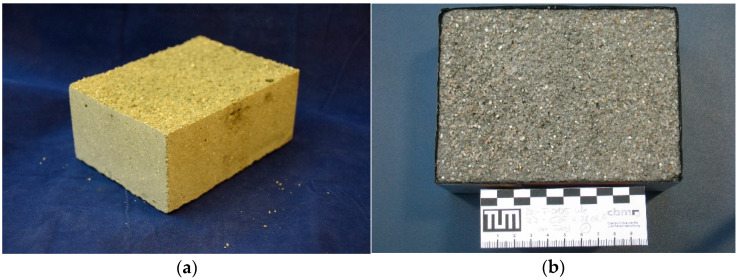
3D printed (90°) specimen with (CDF) and without (CIF) deicing salt (**a**) with already sawn edges before sealing with Teflon tape with untreated test surface and (**b**) sealed with Teflon tape.

The specimens were wet-sawn to a size of the test surface of 150 × 110 mm^2^. The test surface of the SPI specimens exposed to the freeze–thaw impact was the untreated surface resulting from the printing process; see Figure 8. The edges of the casted as well as of the 3D-printed specimens were sealed by a Teflon tape. The storage at 20 °C and 65% RH was followed by 7 d of capillary suction before the specimens were tested for 56 freeze–thaw cycles (28 d).

The scaling (external damage) was determined by the cumulative weight loss of concrete in g/m^2^ over the test period and the internal damage by the decrease of ultrasound speed (rel. dyn. E-Modulus) according to the standard and evaluated according to the German guideline BAW Merkblatt: Frostprüfung von Beton (MFB) [44].

#### 2.3.8. Determination of the Carbonation Resistance

The carbonation resistance was measured in accordance to DIN CEN/TS 12390-10:2007 [45]. The specimens were stored at 20 °C and 65% RH under atmospheric CO_2_ (natural carbonation) and under an increased CO_2_ content of 2 vol % (accelerated carbonation).

The test specimens were either conventionally casted (100 × 100 × 400 mm^3^) or 3D printed (100 × 100 × 200 mm^3^). The specimens were left in the formwork for 1 day or excavated from the particle bed after 1 day; then, they were stored under water until an age of 7 days. Subsequently, the specimens were stored at 20 °C and 65% RH.

In order to speed up the long test procedure for measuring the carbonation resistance, current research is aimed at obtaining faster test results by increasing the CO_2_ concentration; see, among others, Bier [46], Castellote et al. [47], and Thiel et al. [48,49,50].

In consequence, the naturally exposed specimens remained in that climate until the day of testing (age 182 days), the other series of specimens was stored for 28 days in natural exposure, followed by a storage under increased CO_2_ content of 2 vol % under atmospheric pressure for another 28 d to provoke the wanted acceleration of the carbonation reaction [46,49] and were tested with an age of 56 d. When using this method, it must be taken into account that the increased CO_2_ content also causes increased moisture content behind the carbonation front, and a faster expanding substance space leads to an encapsulation of portlandite, which can lead to an overestimation of the carbonation resistance. This can be overcome by applying increased pressure to the sample. However, this is still the focus of ongoing research and therefore not conclusively clarified [48,50].

The specimens were split on the test day, the split surfaces were sprayed with phenolphthalein solution, and the carbonation depth was measured with an accuracy of 0.1 mm.

#### 2.3.9. Determination of Chloride Penetration Resistance

The chloride penetration resistance of concrete has been tested by means of another accelerated test method, which is the rapid chloride migration method (RCM-Method). The rapid chloride migration coefficient D_RCM_ (10^−12^ m^2^/s) was tested following the German guideline BAW Merkblatt Dauerhaftigkeitsbemessung und -bewertung von Stahlbetonbau bei Carbonatisierung und Chlorideinwirkung (BAW Code of Practice—Resistance of Concrete to Chloride Penetration [51]) according to 2012 [52] and 2017 [53], respectively.

**Figure 9:**
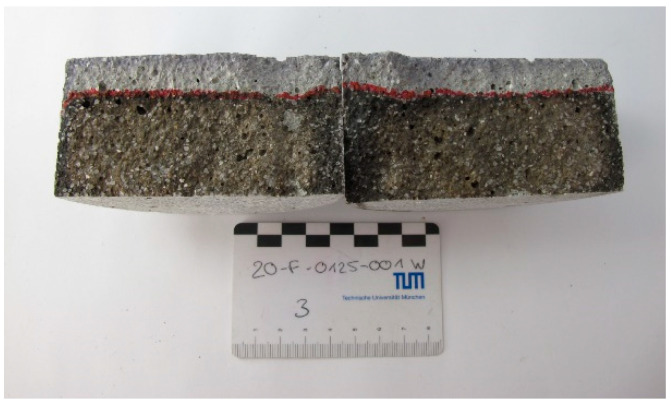
Cross-section of a 3D-printed D_RCM_ specimen (tested in 90°); light gray color on top: area with penetrated chloride; dark gray/brown color below: area without chloride.

In this test method, concrete cylinders with a diameter of 100 mm and a height of 50 mm are installed in a migration cell and subjected to a 10% NaCl solution from below at an age of 28 d (for fast-hardening concretes) or 56 d and to a voltage to accelerate the ion transport. After testing, the specimen is split and sprayed with silver nitrate solution and potassium dichromate solution; see Figure 9.

The areas exposed to chloride show a lighter coloration, and the penetration depth can be determined with a caliper. From this, the rapid chloride migration coefficient D_RCM_ (10^−12^ m^2^/s) is calculated.

For this purpose, test specimens with dimensions 120 × 120 × 70 mm^3^ were prepared parallel (0°) and perpendicular (90°) to the layers in the particle bed. From them, cylinders with a height of 50 mm and a diameter of 100 mm were drilled and sawn. Since the cement is normal-setting cement, the SPI specimens were tested after 28 d.

The reference specimens were obtained from cubes with a side length of 150 mm. Cylinders with a diameter of 100 mm were drilled out. From this cylinder, 10 mm were first cut off from the concreting side. The following section (50 mm) was used as a 28 d D_RCM_ specimen. The next 50 mm section was tested after 56 d.

All specimens were stored in the particle bed or formwork for 1 d and then under water until 28 d or 56 d, respectively. One week before testing, the cylinders were cut to size for testing. For the test, the shell surface of the specimens was sealed by insertion into a fabric-reinforced rubber hose and a hose clamp to prevent lateral ingress of the test liquids (top face of the cylinder: Potassium hydroxide solution, bottom face of the cylinder: 10% sodium chloride solution in potassium hydroxide solution).

A summary of the strength and durability measurements can be found in Appendix B, Table A2.

## 3. Results and Discussion

### 3.1. Compressive and Flexural Strength

**Figure 10:**
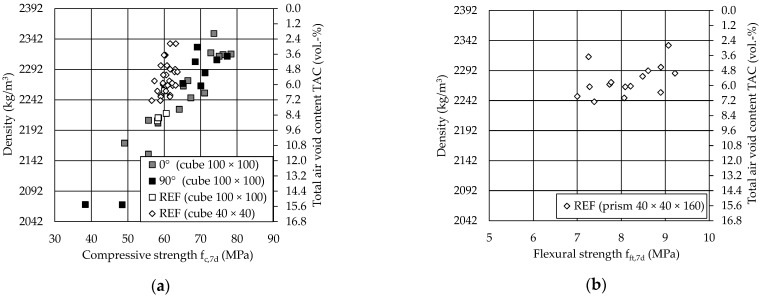
Strength values after 7 d: (**a**) Compressive strength and (**b**) Flexural strength.

Figure 10a shows the compressive strength after 7 d of the printed specimens in comparison with the results of the reference specimens as a function of the density and the total air void content (TAC).

The compressive strength increases with the density and with decreasing total air void content. The SPI specimens achieve even higher compressive strengths with the same density or TAC compared to the reference prisms, although the prism specimens had a smaller size. This can be explained by the different manufacturing processes. Due to the mixing of the cement paste and the aggregates for the reference specimens, there is probably an initial layer of cement paste around almost all particles. The cement paste layer can only partly be displaced into the voids between the particles by compaction, and presumably, an only incomplete grain structure is formed. In the SPI manufacturing process, there is a direct grain contact and therefore a grain structure before the cement paste penetrates between the voids. Therefore, the grain structure of the SPI specimens can transmit equal or larger loads even at lower filling ratios of the voids in the particle bed, since the cement paste plays a subordinate role in the force transmission. This is also confirmed by the fact that the 7 d values of the SPI specimens already show the same or higher strengths as the 28 d values of the reference specimens [29].

Furthermore, it can be seen that the test direction 0° or 90° does not seem to have any effect on the compressive strength, even with smaller filling ratios of the voids. This could again be explained by the force transmission via the grain structure, in which the cement paste mainly serves to stabilize the grain structure as soon as the layers are completely penetrated (penetration depth ≥ the layer thickness).

The specimens achieve strengths between 68 MPa and 78 MPa with a TAC of 2–7 vol %, see also [28]. Thus, at comparable density (2100 kg/m^3^), higher compressive strengths can be achieved comparing to [54] (37.3 MPa perpendicular to the layers, 90° and 33.8 MPa parallel to the layers, 0°).

Figure 10b exhibits the flexural strength of the reference specimens (prims with 40 × 40 × 160 mm^3^) after 7 d. The material achieves a flexural strength of up to 9.2 MPa with a slight dependency on the density. The quite high values of the flexural tensile strength (≈15% of the compressive strength) without the use of reinforcement should be emphasized but lie in the expectable range of 1/10 of the compressive strength.

Due to the comparable results of the compressive strength for the 3D-printed and reference specimens, the determination of the flexural strength for the 3D-printed specimens was not performed for this paper. However, especially the flexural strength could be more susceptible for an anisotropic material behavior and should therefore be investigated in future test series.

### 3.2. Freeze–Thaw Resistance

#### 3.2.1. Scaling

**Figure 11:**
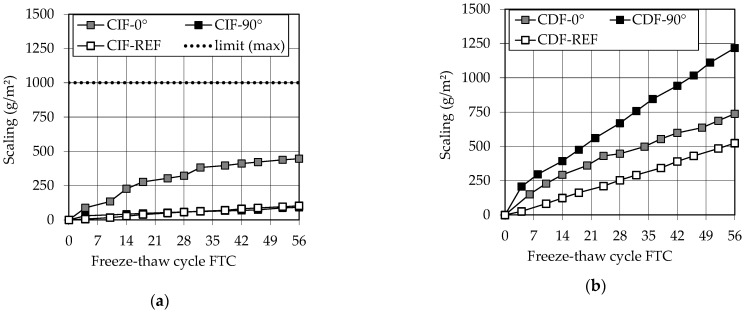
Scaling of (**a**) CIF specimens and (**b**) CDF specimens.

Figure 11 shows the cumulative scaling (weight loss) in g/m^2^ over the number of freeze–thaw cycles (FTW) for (a) CIF specimens (without de-icing salt) and (b) CDF specimens (with de-icing salt) as a measure for the external damage.

In Figure 11a, the printed specimens loaded in 90° to the printing layer and the reference specimens show only very low scaling of less than 125 g/m^2^ after 56 days. The specimens loaded in 0° to the layers exhibit a higher scaling of up to 450 g/m^2^ after the test period. However, all specimens remain well below the required acceptance criterion of maximum 1000 g/m^2^ after 28 freeze-thaw cycles (FTC) according to the German guideline BAW-Merkblatt: Frostprüfung von Beton (MFB) [44]. The specimens also do not show a noticeable anisotropic behavior. The deviation of the 0° specimens in the scaling can be neglected, since this lies within the coefficient of variation of the measurement method of ν = 32% in the scaling range 0 to 500 g/m^2^ according to [44]. In addition, only two individual specimens could be tested per 3D-printed series due to the limitations in the construction space. In order to be able to make more reliable statements, further series should be tested.

Furthermore, all test series loaded with de-icing salt (CDF, Figure 11b) show a scaling below the limit value of 1500 g/m^2^ required by [44] after 28 FTC as well as still after 56 FTC. At 670 g/m^2^, the scaling after 28 FTC is the highest for the in 90° exposed test surface of the printed specimens (black squares). At the same time, the reference specimens (white squares) show the lowest scaling of 255 g/m^2^ after 28 FTC. This reverses the scaling results of the CIF test, where the specimens exposed in 0° to the layers showed higher scaling than the specimens exposed in 90°. However, the measurement inaccuracies are also within the coefficient of variation of ν = 17.5% according to [44]. Thus, again, no statement can be made about a real anisotropy of the results.

These good results can presumably be attributed to the low w/c ratio of 0.3 and the TAC of the specimens ranging from 4.0 vol % to 5.6 vol %, since a w/c ratio of 0.55, 0.50, and 0.45, respectively, and a minimum TAC of 4.0 vol % are required for exposure classes XF2, XF3, and XF4 according to EN 206:2013+A1:2016 [55].

**Figure 12:**
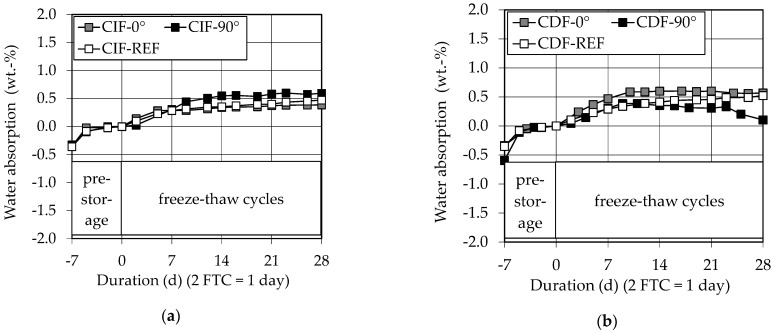
Water adsorption of (**a**) CIF specimens and (**b**) CDF specimens.

The good results of the scaling during the freeze–thaw tests and the assumption of a dense pore structure combined with a sufficient TAC can be confirmed by the low water absorption during the pre-storage and the freeze–thaw cycles, see Figure 12a,b of maximum 0.6 wt %.

#### 3.2.2. E-Modulus

**Figure 13:**
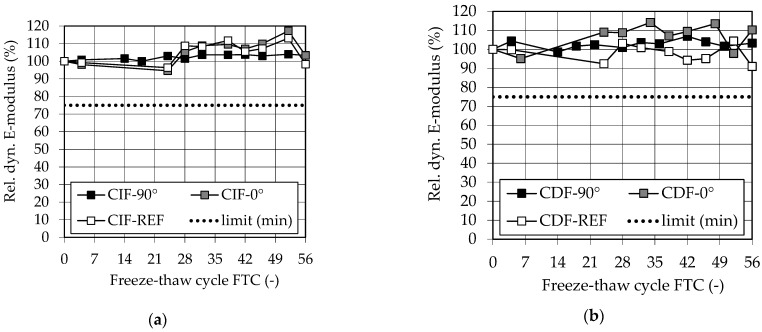
Rel. dyn. E-modulus of (**a**) CIF specimens and (**b**) CDF specimens.

Figure 13 shows the relative dynamic E-modulus as a measure for the inner damage of (a) specimens exposed to water (CIF) and (b) specimens exposed to de-icing salt (CDF).

The measurement of the relative dynamic E-modulus, which is decisive for the CIF test, shows no internal damage (acceptance criteria: rel. dyn. E-modulus ≥75%) in any of the series after 28 FTC and even after 56 FTC according to [44] for the CIF and CDF test samples.

The missing measuring points of the reference specimens and the 0° specimens on three measuring days are conspicuous, which were caused by a defect in the measuring device and had to be corrected. The deviation of the measuring points and the increase of the values up to almost 120% in some cases are also unusual. This is probably also due to the change of the measuring device during the test series. However, also for the inner damage, no anisotropic behavior can be observed.

Furthermore, the good results can be explained by the material composition and the TAC of the specimens (see Section 3.2.1).

### 3.3. Carbonation Resistance

As Table 1 shows, neither the naturally carbonated specimens after 182 d nor the specimens stored under elevated CO_2_ content of 2 vol % after 56 d (28 d of exposure) exhibit any detectable carbonation.

The good results can be explained by the high calcium hydroxide (CH) content due to the cement type (OPC—CEM I 42.5R) as well as the dense pore structure due to the w/c ratio of 0.3, which thus reduces the carbonation progress. In addition, the presumably reduced carbonation depth due to the increased CO_2_ concentration must be taken into account [46,48].

These first investigations on the carbonation resistance of the test specimens show that they can probably be used for exterior components with an exposure class XC4 [55]. With the help of [53], the carbonation depth can be predicted for a given service life of the structure, and thus the minimum concrete cover can be determined for the use of steel reinforcement if the location is known.

However, due to the short observation period of 182 d (recommended observation period according to [45] 182 d–730 d) under atmospheric conditions and the small number of samples, further investigations must be carried out.

### 3.4. Chloride Penetration (Migration) Resistance

**Figure 14:**
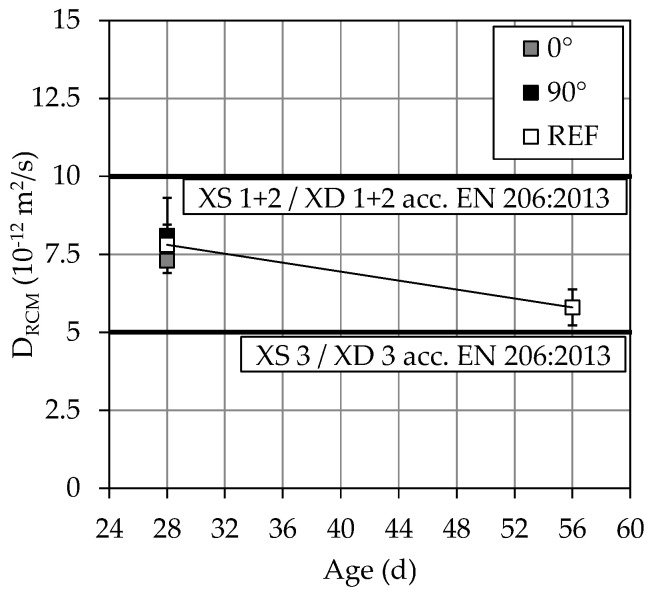
Chloride migration coefficient D_RCM_.

Figure 14 shows the rapid chloride migration coefficient D_RCM_ determined and evaluated after 28 days and 56 days, respectively.

The 3D-printed specimens and the reference specimens show similar results after 28 d and achieve, following [51,52], an exposure class of XS2 and XD2 according to EN 206:2013 + A1:2016 [55]. Furthermore, the 3D-printed specimens seem to exhibit isotropic material behavior. The reference specimens even almost meet the requirements for exposure class XS3 and XD3 after 56 d. Since the material of the reference as well as the 3D-printed specimens is the same, it can be assumed that the SPI specimens would have shown similar results.

According to the author’s current knowledge and also according to Rahimi [56], no comparative D_RCM_ values can be found in the literature for mortars or concretes with a w/c ratio of 0.3 made from OPC (CEM I). However, for a w/c ratio of 0.4 and OPC (CEM I) in Krishnakumar et al. [57], a D_RCM_ of ~9.2∙10^−12^ m^2^/s as well for OPC (CEM I 42.5 R) in Gehlen [58], a D_RCM_ = 8.9∙10^−12^ m^2^/s was determined. Furthermore, both references show that the D_RCM_ becomes lower with decreasing w/c ratio due to the finer pore structure, which confirms the lower D_RCM_ values between 7.3∙10^−12^ and 8.1∙10^−12^ m^2^/s in Figure 14. In addition, at comparable water to binder (w/b) ratios, much lower D_RCM_ values could be expected from the use of binder compositions containing silica fume [59,60].

However, it must be noted that only three specimens were investigated per series. In the future, further investigations of the D_RCM_ must be carried out to confirm the results.

## 4. Conclusions

This paper presents measurements of the strength and durability of SPI printed components in dependency of layer orientation compared to casted specimens with the same mixing composition. The results showed the following main findings:

- All results exhibited in almost all cases no dependency on the layer orientation.
- Casted and 3D-printed specimens showed comparable results.
- Maximum compressive strength values up to 78 MPa after 7 d.
- Flexural strength of 1/10 of compressive strength.
- High freeze–thaw resistance.
- No detectable carbonation depth after 182 days of natural CO_2_ exposure and after 28 days under increased CO_2_ content of 2 vol %.
- Low rapid chloride migration coefficients, D_RCM_.

However, due to the limited building space of the printer, some measurements are based on only a few results. Therefore, more tests should be carried out to confirm the findings of this research.

## Figures and Tables

**Table 1:** Carbonation depths of the test series.

Series	Carbonation Depth for Atmospheric CO_2_ Content (Age 182 d)	Carbonation Depth for 2 vol % CO_2_ Content (Age 56 d)
-	mm	mm
3D printed (0°)	0.0 (none of the three test series exhibited visual carbonation)	
3D printed (90°) REF		

## Data Availability

Data sharing is not applicable to this article.

## Appendix A

The yield stress and the viscosity were determined in a rotational double-plate measuring system (diameter 50 mm, gap distance 1 mm, rheometer: Anton Paar MCR 502) 900 s after addition of the mixing water. The plates had a roughness with a depth of 0.5 mm to prevent wall slip. The profile started with an average shear rate of 40 s^−1^ for 10 s to achieve a complete structural break up (for the given shear history). Afterwards, the measurement was carried out with 19 descending steps of 80 s^−1^ to 0.02 s^−1^ with a step duration of 6 s. Preliminary tests showed that with this profile, an equilibrium (an approximately constant value of the shear stress in every step) could be achieved in the steps between 60 and 2.5 s^−1^ for all measured pastes. Below 2.5 s^−1^, the effect of thixotropy was already recognizable by a rising value of the shear stress.

The yield stress and the viscosity were calculated using the model of Herschel–Bulkley, which is a common model to describe the flow behavior of cement pastes [61,62,63,64,65,66], see Equations (A1) and (A2).

(A1) $$\tau =\tau_{0,\mathrm{HB}}+k\cdot {\dot{\gamma }}^{n},$$

τ_0,HB_ [Pa] is the (Herschel–Bulkley) yield stress and k [Pa∙s^n^] is the consistency factor. In addition, the flow index n describes a shear-thinning behavior in the range 0 < n < 1 and a shear-thickening behavior in the range 1 < n < ∞. If n = 1, it becomes a Bingham fluid.

The viscosity of Herschel–Bulkley is always given as a function of shear rate. Consequently, the shear rate-dependent, (Herschel–Bulkley) viscosity η($\dot{\gamma }$) (Pa∙s) is calculated according to Equation (A2).

(A2) $$\eta \left(\dot{\gamma }\right)=\frac{\tau_{0,\mathrm{HB}}}{\dot{\gamma }}+\;k\cdot {\dot{\gamma }}^{n-1}$$

The thixotropy was determined with a rotational vane-in-cup geometry (radius cup 35 mm, height vane blade 60 mm, radius vane geometry 20 mm, number of blades 6, rheometer: Anton Paar MCR 502). The vane cup had rips to prevent wall slip. At first, the cement paste was mixed with 90 rpm for 30 s in order to remove the internal structural build-up in the cement paste, which could have been formed during end of mixing and installation of the vane cup in the rheometer. Then, 30, 60, 120, and 240 s after completion of the structural break-up, the static yield stress τ_0,s_ was determined at 0.094 rpm. This measurement speed was identified in preliminary tests as the speed that could just cause a structural breakup.

Thus, τ_0,s_ is the maximum shear stress (structural break-up) on the outside of the vane geometry at every measurement time. A_Thix_ (Pa/s) can be determined as the slope of the static yield stress assuming a linear development of the static yield stress over time [66]; see Equation (A3).

(A3) $$A_{\mathrm{Thix}}=\frac{\tau_{0,s}\left(t\right)-\tau_{0,\;\mathrm{HB}}}{t_{P}}\;,$$

where t_P_ (s) is the corresponding time period.

**Table A1:** Properties of the cement paste.

w/c Ratio	Mini Slump Flow	Density ρ_P_	Yield Stress τ_0_	Consistency k	Flow Index n	Thixotropy
-	mm	kg/m^3^	Pa	Pa∙s^n^	-	Pa/s
0.30	400	2090	2.7	0.12	1.26	0.42

## Appendix B

**Table A2:** Summary of strength and durability measurements.

Test Method	Number of Samples 90°/0°/REF	Age at Testing (Begin)	Procedure Acc. to Standard
-	-	d	-
Compressive strength	10/14/35	7	DIN EN 12390-3:2009-07 [41]/ DIN EN 196-1:2016-11 [42]
Flexural strength	-/-/15	7	DIN EN 196-1:2016-11 [42]
Freeze–thaw resistance (CIF)	2/2/5	35 (after 7 d pre-storage)	DIN CEN/TS 12390-9:2017-05 (DIN SPEC 91167) [43]
Freeze–thaw resistance (CDF)	2/2/5	35 (after 7 d pre-storage)	DIN CEN/TS 12390-9:2017-05 (DIN SPEC 91167) [43]
Carbonation resistance (ambient conditions)	3/3/3 (90° and 0° were tested on the same sample)	182	DIN CEN/TS 12390-10:2007 [45]
Carbonation resistance (2 vol % CO_2_)	3/3/3 (90° and 0° were tested on the same sample)	28	-
Chloride penetration resistance	3/3/6	28	German guideline BAW Code of Practice—Resistance of Concrete to Chloride Penetration [51,52,53]
